# Pharmacokinetics and safety of IBI301 versus rituximab in patients with CD20^+^ B-cell lymphoma: a multicenter, randomized, double-blind, parallel-controlled study

**DOI:** 10.1038/s41598-020-68360-0

**Published:** 2020-07-15

**Authors:** Bo Jiang, Xiaoyan Ke, Qingyuan Zhang, Wei Xu, Hang Su, Jie Huang, Mingzhi Zhang, Huaqing Wang, Chuan Jin, Jun Zhu, Li Liu, Zhen Cai, Xielan Zhao, Jianfeng Zhou, Xiaohong Zhang, Jing Liu, Hui Zhou, Jie Yu, Xing Sun, Junyuan Qi, Lugui Qiu

**Affiliations:** 1 State Key Laboratory of Experimental Hematology, National Clinical Research Center for Hematological Disorders, Institute of Hematology and Blood Diseases Hospital, Chinese Academy of Medical Sciences and Peking Union Medical College, Tianjin, China; 2 Hematology Department, Peking University Third Hospital, Beijing, China; 3 Oncology Department, The Affiliated Cancer Hospital of Harbin Medical University, Harbin, China; 4 Hematology Department, Jiangsu Province Hospital, Nanjing, China; 5 Department of Lymphoma/Head and Neck Oncology, The Fifth Medical Center of PLA General Hospital, Beijing, China; 6 Hematology Department, West China Hospital Sichuan University, Chengdu, China; 7 Oncology Department, The First Affiliated Hospital of Zhengzhou Medical University, Zhengzhou, China; 8 Oncology Department, Tianjin Union Medical Center Nankai University Affiliated Hospital, Tianjin, China; 9 Forth Medical Department, The Affiliated Cancer Hospital of Guangzhou Medical University, Guangzhou, China; 10 Key Laboratory of Carcinogenesis and Translational Research (Ministry of Education), Department of Lymphoma, Peking University Cancer Hospital and Institute, Beijing, China; 11 Hematology Department, Tangdu Hospital, The Medical University of Air Forces, Xi’an, China; 12 Bone Marrow Transplantation Center, The First Affiliated Hospital of Zhejiang University School of Medicine, Hangzhou, China; 13 Hematology Department, Xiangya Hospital Central South University, Changsha, China; 14 Hematology Department, Tongji Medical College Huazhong University of Science and Technology, Wuhan, China; 15 Hematology Department, The Second Affiliated Hospital of Zhejiang University School of Medicine, Hangzhou, China; 16 Hematology Department, The Third Xiangya Hospital of Central South University, Changsha, China; 17 Innovent Biologics, Inc., Suzhou, China

**Keywords:** Lymphoproliferative disorders, Immunological disorders

## Abstract

This multicenter, randomized, double-blind, parallel-controlled trial aimed to compare the pharmacokinetics (PK) of IBI301 with rituximab in patients with CD20-positive (CD20^+^) B-cell lymphoma, who achieved a complete response/unconfirmed complete response after standard treatments. Patients were randomized (1:1) to receive IBI301 or rituximab (375 mg/m^2^, IV). Patients who continuously benefitted from the trial after the PK phase underwent the extension phase to receive up to three cycles of 3-month-cycle of rituximab/IBI301 maintenance therapy. PK was described using the area under the serum concentration–time curve from time zero to infinity (AUC_0-inf_), AUC from time zero to last quantifiable concentration (AUC_0-t_), and maximum serum concentration (*C*_max_). Pharmacodynamics (PD), incidence of adverse events and immunogenicity were evaluated. PK was defined equivalent, if 90% confidence intervals (CIs) for geometric mean ratios of PK endpoints fell within the margin of 0.8–1.25. Overall, 181 patients were enrolled in IBI301 (*n* = 89) and rituximab (*n* = 92) groups. Geometric mean ratios of AUC_0-inf_, AUC_0-t_, and *C*_max_ were 0.91 (90% CI 0.85, 0.97), 0.91 (90% CI 0.86, 0.97), and 0.96 (90% CI 0.92, 1.01) between treatment groups, all within the bioequivalence range. Peripheral CD19^+^ and CD20^+^ B-cell counts were similar at each prespecified time point between the groups. No difference in immunogenicity was observed. The incidences of treatment-emergent adverse events (84.3% vs. 83.5%) and treatment-related AEs (56.2% vs. 61.5%) were comparable (IBI301 vs. rituximab). IBI301 was PK bioequivalent to rituximab in patients with CD20^+^ B-cell lymphoma. The PD, safety, and immunogenicity profiles of IBI301 were similar to those of rituximab.

## Introduction

Non-Hodgkin lymphoma (NHL) is the seventh most common cancer worldwide, accounting for 4–5% of all new cancer cases and 3–4% of cancer-related deaths^1^. Diffuse large B-cell lymphoma (DLBCL) is the most common histologic subtype of NHL, which is quite heterogeneous in terms of morphology, genetics, and biologic actions^2^.

Cluster of differentiation (CD) 20 is involved in the proliferation of cancerous B cells, and anti-CD20-based immuno-chemotherapy is the standard first-line treatment for NHL^3,4^. CD20-positive (CD20^+^) NHLs respond to anti-CD20 therapies such as rituximab, which has improved the management of NHL^4^. Rituximab is an engineered chimeric mouse/human monoclonal immunoglobulin G1κ antibody against the CD20 antigen of B cells^5^. As of 2012, more than 3 million patients worldwide have been treated with rituximab^6^. A real-world study of patients treated between 2011 and 2016 in China showed that rituximab plus chemotherapy as the first-line treatment for DLBCL had an objective response rate of 94.2%^7^.

Unfortunately, access to biological products such as rituximab is limited, despite a huge clinical need. Alternatively, biosimilar is a preferable choice when the original patent expires (for rituximab, in 2013 in Europe and in 2018 in the United States)^5,8^. A biosimilar is defined as a biological product that has a high similarity in terms of efficacy, potency, and safety to an approved biologic product (originator)^9–11^, and is less expensive^12^. A number of potential rituximab biosimilars are under development^5,13^. Among them, CT-P10 has been approved for the treatment of NHL and rheumatoid arthritis (RA) in South Korea and Europe^13^. The development of rituximab biosimilars increases patient access to this reference drug. IBI301 is a candidate rituximab biosimilar under development jointly by Innovent Biologics, Inc. (Suzhou, China) and Eli Lilly and Company (Indiana, USA). A study with a small sample size of Chinese patients showed that IBI301 monotherapy was well tolerated in patients with CD20^+^ NHL^14^.

The phase 2 study aimed mainly to compare pharmacokinetics (PK) between IBI301 and rituximab in patients with CD20^+^ B-cell lymphoma who achieved a complete response/unconfirmed complete response (CR/CRu) after standard treatments. The secondary purposes were to evaluate the AUC from time zero to last quantifiable concentration (AUC_0-t_), the maximum serum concentration (*C*_max_), pharmacodynamics (PD) endpoints, safety, and immunogenicity profiles of IBI301 compared with rituximab.

## Methods

### Study design and participants

This was a multicenter, randomized, double-blind, parallel-controlled phase 2 study (NCT02945215), conducted in 17 sites in China under the guidance of the ethical and scientific principles of the Declaration of Helsinki and Good Clinical Practice. Since rituximab targets CD20 and causes immunological cytolysis^5,15,16^, only patients with CD20^+^ B-cell lymphoma were enrolled.

The major inclusion criteria for participants were as follows: (1) histologically or cytologically confirmed CD20^+^ B-cell lymphoma; (2) achieved a CR/CRu according to the standard response criteria for NHL (International Working Group 1999 criteria)^17^ after standard treatment (including rituximab plus chemotherapy, chemotherapy alone, or other therapies); a CRu was confirmed by enhanced computed tomography or magnetic resonance imaging; (3) patients aged between 18 and 65 years; (4) Eastern Cooperative Oncology Group performance status 0 or 1; (5) at least 6 months of life expectancy as evaluated by investigators; and (6) previous anti-tumour therapies terminated (at least 4 weeks for chemotherapy, 16 weeks for rituximab or other targeted therapies, and 4 weeks for radiotherapy). Adverse events (AEs) associated with chemotherapy, surgery, radiotherapy, or targeted therapy had been alleviated to no more than 1 grade. The key exclusion criteria were as follows: (1) received transfusion, erythropoietin, granulocyte colony-stimulating factor (G-CSF), or granulocyte–macrophage CSF treatments within 14 days prior to enrolment; (2) received high-dose corticosteroid treatment (prednisolone > 10 mg/day or equivalent dose of other drugs) 28 days prior to enrolment; or (3) allergic or hypersensitive to rituximab or any other monoclonal antibodies (mAbs). Patients with a residual concentration of peripheral rituximab > 24 μg/mL were also excluded, however, they were allowed a re-selection after a washout period when the concentration of peripheral rituximab was lower than 24 μg/mL. The detailed inclusion and exclusion criteria are provided in the Supplementary information.

### Procedures

Eligible patients were randomized to either IBI301 or rituximab group (1:1) using an interactive web response system, stratified by previous rituximab use and CR/CRu status upon enrolment. In this double-blind study, the study drugs were repackaged in a unified way. Except for dispensing nurses, the investigators, subjects, independent review committee, and data analysts were all blind to treatment allocation.

According to the pre-clinical data of IBI301, and the clinical dose recommendation (375 mg/m^2^) of rituximab (HoffmaNN-La Roche, BASEL, Switzerland) for lymphoma treatment, the IBI301 dose was set at 375 mg/m^2^. The study included two phases: PK phase and extension phase. In the PK phase, each participant received once IBI301 or rituximab (375 mg/m^2^) intravenously; PK, PD, safety, and immunogenicity were evaluated 13 weeks after drug administration. The extension phase was performed for the sake of ethics to ensure the interest of patients who could continuously benefit from the drugs under the judgment of investigators. During the extension phase, the participants received IBI301 or rituximab (375 mg/m^2^) maintenance therapy, once every 3 months, and up to three infusions.

### PK and PD analyses

Blood samples were collected for all participants at the pre-determined 11 time points: 1 h before infusion, within 5 min immediately after infusion, and 6 h, 24 h, 72 h, 168 h (8 days), 14 days, 28 days, 42 days, 70 days, and 91 days after infusion. The concentrations of IBI301 (Innovent Biologics, Inc, Suzhou, China) and rituximab (Shanghai Roche, Shanghai, China) were measured by enzyme-linked immunosorbent assay using a human mAb kit (Bio-Rad, California, USA) specific to rituximab/IBI301. In brief, a solid-phase 96-well microtiter plate was coated with human mAb (1 μg/mL) specific to rituximab and incubated with 200 μL of the blocking buffer (Kirkegaard and Perry Laboratories [KPL], Gaithersburg, MD, USA) in each well at 2–8 °C overnight. Then the serum samples containing IBI301 or rituximab (100 μL/well) were added. Subsequently, the serum samples were incubated with the secondary antibody of horseradish peroxidase (HRP)–conjugated rat-human rituximab antibody (1:5,000) (Bio-Rad, California, USA) for 90 ± 10 min. A solid-phase carrier–IBI301/rituximab–enzyme complex was formed after incubation of the secondary antibody with IBI301 or rituximab. Then, the 3,3′,5,5′-tetramethylbenzidine membrane substrate (KPL, Gaithersburg, MD, USA) (100 μL) of HRP was added to each well, and the complex was incubated for 15 ± 5 min at room temperature. Further, 1 N sulfuric acid (50 μL/well) (Sinopharm, Shanghai, China) was added to stop the reaction, and SpectraMax (Molecular Devices, San Jose, USA) was used to measure the absorbance value at 450 nm (reference 650 nm) within 15 min after quenching.

The percentage and absolute count of peripheral CD19^+^ and CD20^+^ B cells were measured using flow cytometry, within 1 h before infusion, and 72 h, 28 days, and 91 days after infusion.

### Immunogenicity

Blood samples were collected from all participants during the screening period, and 14 days, 28 days, 42 days, 70 days, and 91 days after infusion. The anti-drug antibody (ADA) analysis was performed using the electrochemiluminescence method (Supplementary information). The ADA-positive serum samples were further tested for neutralizing antibodies (NAb) (Supplementary information).

### Safety

The safety assessment included mainly AEs and immunogenicity. Data on AEs were collected from the signing of the informed consent form to 3 months after the last infusion in the extension phase. Treatment-emergent AEs (TEAEs) were defined as all the AEs occurring from the initiation of the study to the last follow-up time, and the treatment-related AEs (TRAEs) were defined as AEs associated with the use of the study drug, as determined by the investigators. AEs and laboratory abnormalities were recorded and classified according to the National Cancer Institute Common Terminology Criteria for Adverse Events (V4.03). The AEs were coded using MedDRA (version 16.1 or above).

### Assessments

PK was described using the area under the serum concentration–time curve from time zero to infinity (AUC_0-inf_), AUC_0-t_, and *C*_max_. The primary endpoint was AUC_0-inf_, and the secondary endpoints were AUC_0-t_, *C*_max_, PD parameters of the absolute counts and percentages of peripheral CD19^+^ and CD20^+^ B cells at each prespecified time point, and positive rates of ADA and NAb. In addition, clearance (CL) and half-life (*T*_1/2_) were also analysed.

### Statistical analysis

The PK bioequivalence was considered if 90% confidence intervals (CIs) for the ratio (IBI301/rituximab) of geometric means of the primary endpoint fell within the bioequivalence margin of 0.8–1.25. Assuming the ratio of the geometric means of the AUC_0-inf_ was between 0.95–1.05 and the coefficient of variation (CV) was 35%, the minimum sample size was estimated to be 112 to obtain a power of 80% with a significant level of *α* = 0.05 (two one-sided). Therefore, 140 patients were planned to be enrolled (70/group), allowing a 20% dropout rate. After reviewing the AUC data of the first 70 participants, the sample size was re-estimated based on the actually observed CV and the ratio of AUC, and increased to 180. During the interim analysis, the Peto-Prentice test was used to adjust *α*^18,19^ and a nominal level *α* = 0.001 (one-sided) was used. During the final analysis, *α* = 0.05 (one-sided) was used to perform the statistical analysis.

The safety analysis set (SS) included the patients who received at least one dose of the study drug. The PK analysis set (PKS) included the patients from the SS who had no major protocol deviation and no missing data of serum sample collection.

The PK parameters were estimated based on a non-compartmental model, and a linear regression model was used to fit the log-transformed parameter, with the treatment, previous rituximab use and CR/CRu status at baseline as factors in the model.

For PD analysis, the percentage/absolute count of peripheral CD19^+^ and CD20^+^ B cells and the mean difference between the groups with 90% CIs were calculated at different time points. The PD indicators at each time point after administration were calculated and compared with the baseline. The differences between the two groups were compared using the independent 2-group *t* test or the Wilcoxon rank-sum test, based on the distribution as determined using the Shapiro–Wilk test.

Data analyses were carried out using SAS version 9.2 (SAS Institute, NY, USA). A *P* value less than 0.05 were considered statistically significant.

### Ethics approval and informed consent

The study protocol was approved by independent ethics committees at each study center (Ethics Committee of Chinese Academy of Medical Sciences and Peking Union Medical College; Ethics Committee of Peking University Third Hospital; Ethics Committee of the Affiliated Cancer Hospital of Harbin Medical University; Ethics Committee of Jiangsu Province Hospital; Ethics Committee of the Fifth Medical Center of PLA General Hospital; Ethics Committee of West China Hospital Sichuan University; Ethics Committee of the First Affiliated Hospital of Zhengzhou Medical University; Ethics Committee of Tianjin Union Medical Center, Nankai University Affiliated Hospital; Ethics Committee of the Affiliated Cancer Hospital of Guangzhou Medical University; Ethics Committee of Peking University Cancer Hospital and Institute; Ethics Committee of Tangdu Hospital, the Medical University of Air Forces; Ethics Committee of the First Affiliated Hospital of Zhejiang University School of Medicine; Ethics Committee of Xiangya Hospital Central South University; Ethics Committee of Tongji Medical College Huazhong University of Science and Technology; Ethics Committee of the Second Affiliated Hospital of Zhejiang University School of Medicine; Ethics Committee of the Third Xiangya Hospital of Central South University; and Ethics Committee of Institute of Hematology and Blood Diseases Hospital, Chinese Academy of Medical Sciences). All participants signed a written informed consent form. In addition, all methods were performed in accordance with the relevant guidelines and regulations.

## Results

### Enrolment

**Figure 1:**
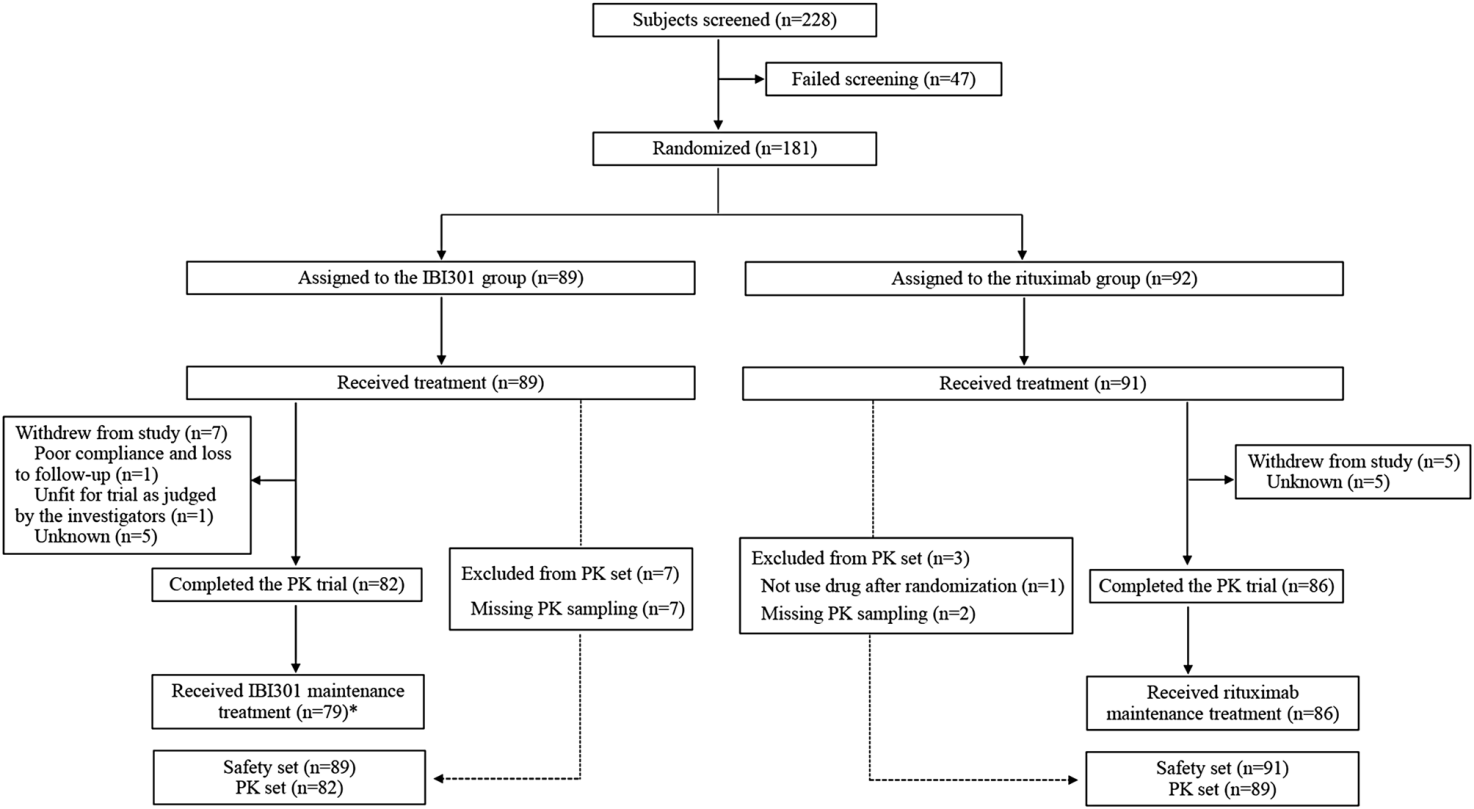
Study flowchart. *PK* Pharmacokinetics.

From December 13, 2016, to October 17, 2018, 181 participants (IBI301 group, *n* = 89; rituximab group, *n* = 92) were enrolled. All participants (100%) in the IBI301 group and 91 (98.9%) in the rituximab group received at least one dose of treatment and were included in the SS. Further, 82 (92.1%) and 89 (96.7%) participants were included in the PKS for the IBI301 and the rituximab group, respectively (Fig. 1). At the data cutoff date on January 14, 2019, 82 (92.1%) participants in the IBI301 group and 86 (93.5%) in the rituximab group completed the PK phase. Seventy-nine (88.8%) participants received IBI301 maintenance treatment in the extension phase, but one participant did not complete the administration. In the rituximab group, 86 (93.5%) participants received rituximab maintenance treatment, and all completed the extension phase. The main disease types were DLBCL (52.8% in the IBI301 group and 48.3% in the rituximab group) and follicular lymphoma (FL) (24.7% in the IBI301 group and 20.9% in the rituximab group). At baseline, a majority of the participants had a stable status achieving a CR, with 79 (88.8%) participants in the IBI301 group and 81 (89.0%) in the rituximab group. The median peripheral rituximab concentration was 2.7 μg/mL and 2.9 μg/mL, respectively, in the two groups. Eighty-seven participants in the IBI301 group and 89 in the rituximab group received prior anti-tumour therapies. Among them, the most common regimen was R ± CHOP ± E (rituximab with/without the chemotherapies of cyclophosphamide, doxorubicin, vincristine and prednisone; and with/without etoposide), which was administered to 81 (93.1%) participants in the IBI301 group and 84 (94.4%) participants in the rituximab group (Supplementary Table S2). Patient baseline demographics and disease characteristics in the SS were well balanced between the two groups (Table 1).

**Table 1:** Demographic and disease characteristics of the patients in the safety set.

	IBI301	Rituximab	All	*P*
	(*n* = 89)	(*n* = 91)	(*n* = 180)	
Age, mean (SD), year	48.5 ± 11.2	49.8 ± 10.5	49.1 ± 10.9	0.41
Sex, *n* (%)				0.66
Male	51 (57.3)	49 (53.8)	100 (55.6)	
Female	38 (42.7)	42 (46.2)	80 (44.4)	
Pathological types, *n* (%)				
DLBCL	47 (52.8)	44 (48.3)	91	–
Follicular lymphoma	22 (24.7)	19 (20.9)	41	–
Marginal zone lymphoma	7 (7.9)	9 (9.9)	16	–
Mantle cell lymphoma	3 (3.4)	5 (5.5)	8	–
High-grade B cell lymphoma	0	2 (2.2)	2	–
Small B cell lymphoma	0	1 (1.1)	1	–
Uncertain subtypes of B cell lymphoma	10 (11.2)	11 (12.1)	21	–
Course of disease, mean (SD), month	19.6 ± 13.3	19.9 ± 13.3	19.7 ± 13.3	0.99
Condition at screening, *n* (%)				
CR	79 (88.8)	81 (89.0)	160 (88.9)	> 0.99
CRu	10 (11.2)	10 (11.0)	20 (11.1)	
Peripheral rituximab, median, μg/mL	2.7	2.9	2.7	0.45
Received prior anti-tumour drug therapy, *n* (%)	87 (97.8)	89 (97.8)	176 (97.8)	> 0.99
Received prior rituximab, *n* (%)	77 (86.5)	79 (86.8)	156 (86.7)	> 0.99
Previous anti-tumour treatment lines, *n* (%)				0.63
1	59 (67.8)	63 (70.8)	122 (69.3)	
2	16 (18.4)	16 (18.0)	31 (17.6)	
≥ 3	12 (13.5)	10 (11.0)	22 (12.2)	
Radiotherapy history, *n* (%)	14 (15.7)	11 (12.1)	25 (13.9)	0.52

*CR* complete response, *CRu* unconfirmed complete response, *DLBCL* diffuse large B-cell lymphoma.

### Pharmacokinetics

**Figure 2:**
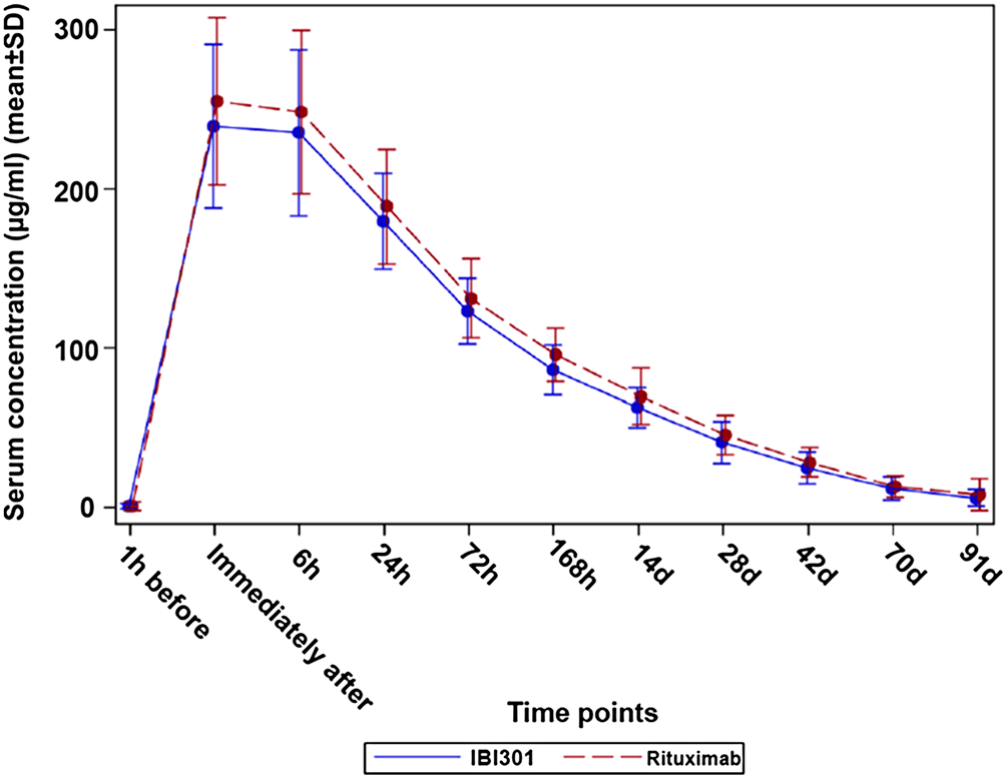
Linear graph of the serum concentrations of IBI301 (blue solid line) and rituximab (red dashed line) over time (μg/mL). Pharmacokinetics analysis set.

**Figure 3:**
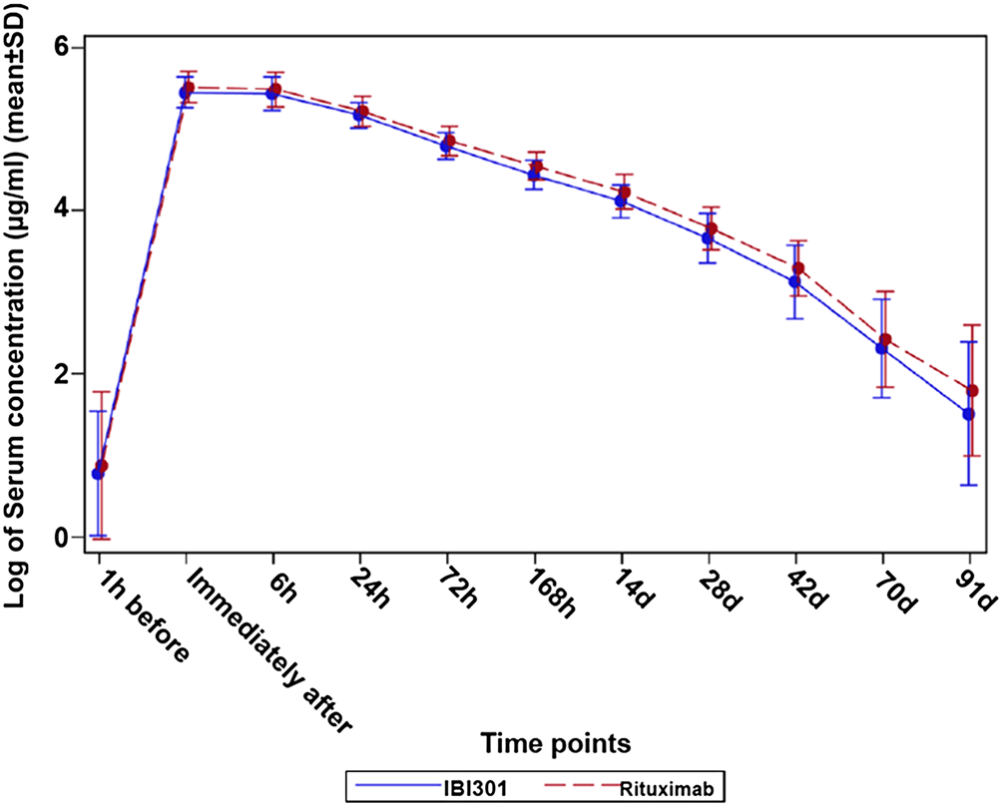
Log10 graph of the serum concentrations of IBI301 (blue solid line) and rituximab (red dashed line) over time (μg/mL). Pharmacokinetics analysis set.

IBI301 and rituximab showed a similar area under drug concentration–time curve (Figs. 2, 3). A rapid decrease in serum concentrations was observed 6 h after infusion, indicating the distribution of the drug in the extravascular compartment. The elimination phase was relatively slow, possibly due to interaction with the target.

The bioequivalence analysis showed that in the PKS, the geometric mean ratio of IBI301/rituximab for AUC_0-inf_ was 0.91 (90% CI 0.85, 0.97), falling within the predetermined acceptable bioequivalent margin of 0.8–1.25. The geometric mean ratios of IBI301/rituximab for AUC_0-t_ and *C*_max_ were 0.91 (90% CI 0.86, 0.97) and 0.96 (90% CI 0.92, 1.01), respectively (Table 2), which also fell within the bioequivalence range. The other PK parameters were also similar between the two groups (Table 3). The PK profiles of IBI301 were basically the same as those of rituximab, and the drug concentrations versus time were comparable between the two groups (Figs. 2, 3).

**Table 2:** Comparison of the PK primary and secondary endpoints in the PK set.

	IBI301 (*n* = 82)	Rituximab (*n* = 89)	Geometric mean ratio (IBI301/rituximab) and 90% CI
AUC_0-inf_ (day × µg/mL)	3,420 (978)	3,769 (1,005)	0.91 (0.85, 0.97)
AUC_0-t_ (day × µg/mL)	3,226 (821)	3,534 (825)	0.91 (0.86, 0.97)
*C*_max_ (µg/mL)	257 (58)	266 (55)	0.96 (0.92, 1.01)

Data are expressed as mean ± SD.

*AUC* area under the curve, *C*_*max*_ maximum serum concentration, *PK* pharmacokinetics.

**Table 3:** PK parameters of the patients in the PK set.

	IBI301 (*n* = 82)	Rituximab (*n* = 89)
*λ*_z_ (1/day)	0.033 (30.8%)	0.032 (29.4%)
*t*_1/2_ (day)	21.1 (30.8%)	22.0 (29.4%)
CL (mL/day)	190 (30.8%)	171 (30.6%)
*V*_SS_ (mL)	5,340 (19.4%)	4,997 (21.6%)
*V*_z_ (mL)	5,801 (20.9%)	5,409 (21.7%)
AUC__Extrap_ (%)	4.138 (115.6%)	4.895 (98.2%)
*T*_max_ (day)^a^	0.181 (0.172, 0.250)	0.194 (0.172, 0.250)

Data are expressed as geometric mean (geometric CV%).

*AUC* area under the curve, *C*_*max*_ maximum serum concentration, *λ*_z_ estimation of the terminal elimination rate constant, *t*_1/2_ half-life, *CL* clearance, *V*_ss_ apparent volume of distribution at steady state, *V*_z_ volume of distribution during terminal phase, *%AUC*_*Extrap*_ area under the curve extrapolated from time t to infinity as a percentage of the total area under the curve, *T*_*max*_ time to reach the maximum concentration.

^a^Data are expressed as median (Q1, Q3).

### Pharmacodynamics

In both groups, compared with the baseline, the absolute values of peripheral CD19^+^ B cells dropped after 72 h (− 97.3% and − 95.5%), reached a nadir on day 28 (− 98.4% and − 98.0%), and then recovered slightly on day 91 (− 96.2% and − 97.1%) (Supplementary Figs. S1–S4). Likewise, the change in the absolute values of peripheral CD20^+^ B cells in both groups was similar: rapidly dropped after 72 h (− 99.0% and − 98.6%), and maintained on day 28 (− 98.9% and − 98.8%) and day 91 (− 96.4% and − 98.7%). Similar changes were noted for the percentage of peripheral CD19^+^ B and CD20^+^ B cells (Supplementary Figs. S1–S4).

### Safety

Comparable safety profiles were observed between the two groups. In the SS, TEAEs occurred in 84.3% participants in the IBI301 group and 83.5% participants in the rituximab group. In the PK phase, the incidence of TEAE was 76.4% and 73.6%, respectively, in the two groups. The most frequent TEAEs (IBI301 vs. rituximab) were decreased white blood cell (WBC) count (33.7% vs. 31.9%), decreased neutrophil count (28.1% vs. 27.5%), and upper respiratory tract infection (23.6% vs. 13.2%). TEAEs of grade 3 or worse were reported in 24 (27.0%) and 12 (13.2%) patients in the IBI301 group and rituximab group, respectively. The most common grade 3 or worse TEAEs in the IBI301 and the rituximab groups were decreased neutrophil count (10.1% vs. 5.5%) and WBC count (6.7% vs. 3.3%) (Supplementary Table S1). No AEs leading to death occurred.

The TRAEs were reported in 50 (56.2%) and 56 (61.5%) patients in the IBI301 group and rituximab group, respectively, and the most common TRAEs (IBI301 vs. rituximab) were decreased WBC count (27.0% vs. 23.1%), decreased neutrophil count (22.5% vs. 19.8%), and decreased lymphocyte count (9.0% vs. 4.4%) (Table 4). Two patients in the IBI301 group reported infections, each with lung infection (grade 1–2) and upper respiratory infection (≥ grade 3). Six patients in the rituximab group experienced lung infection (*n* = 1, grade 1–2), urinary tract infection (*n* = 2, ≥ grade 3), and upper respiratory infection (*n* = 3, ≥ grade 3) (Table 4).

**Table 4:** Summary of treatment-related adverse events in safety set.

	IBI301 group (*n* = 89), *n* (%)		Rituximab group (*n* = 91), *n* (%)	
	Any grade	Grade ≥ 3	Any grade	Grade ≥ 3
Any treatment-related AE	50 (56.2)	12 (13.5)	56 (61.5%)	6 (6.6)
Decreased WBC count	24 (27.0)	4 (4.5)	21 (23.1)	2 (2.2)
Decreased neutrophil count	20 (22.5)	9 (10.1)	18 (19.8)	3 (3.3)
Decreased lymphocyte count	8 (9.0)	0	4 (4.4)	1 (1.1)
Elevated ALT level	7 (7.9)	0	7 (7.7)	1 (1.1)
Elevated AST level	4 (4.5)	0	4 (4.4)	0
Elevated γ-GGT level	4 (4.5)	0	1 (1.1)	0
Decreased hemoglobin level	3 (3.4)	1 (1.1)	1 (1.1)	0
Herpes zoster	3 (3.4)	2 (2.2)	1 (1.1)	0
Lung infection	1 (1.1)	0	1 (1.1)	0
Urinary tract infection	0	0	2 (2.2)	2 (2.2)
Upper respiratory tract infection	1 (1.1)	1 (1.1)	3 (3.3)	3 (3.3)

Data are n (%). Treatment-related adverse events (AEs) occurring in ≥ 3% (≥ 1% partly) and grade ≥ 3 AEs occurring in ≥ 2% (≥ 1% partly) of patients in either group are listed.

*ALT* alanine aminotransferase, *AST* aspartate aminotransferase, *GGT* gamma-glutamyltransferase, *WBC* white blood cell.

TRAEs of grade 3 or worse occurred in 12 (13.5%) and 6 (6.6%) patients in the IBI301 group and rituximab group, respectively, and the most common ones were decreased neutrophil count (10.1% vs. 3.3%) and decreased WBC count (4.5% vs. 2.2%) (Table 4).

### Immunogenicity

Immunogenicity was assessed in 180 participants in the SS (IBI301, *n* = 89; rituximab, *n* = 91). The prevalence of ADA incidence was low in both treatment groups. At baseline, ADA was positive in five participants in each group. The ADA–positive incidence was 5.6% and 5.5% in the IBI301 group and rituximab group after administration, respectively. After IBI301 administration, one participant was still ADA–positive, but the remaining four were ADA–negative. NAb was negative in all 10 participants who were ADA–positive at baseline.

## Discussion

IBI301 is a candidate rituximab biosimilar accepted for the review of Biologics License Application to National Medical Products Administration of China. In this study, the PK results strongly suggested that IBI301 was bioequivalent to rituximab in patients with CD20^+^ B-cell lymphoma who had achieved a CR/CRu after the standard treatment. Additionally, the PD, safety, and immunogenicity profiles of IBI301 were consistent with those of rituximab.

The PK results based on the PKS showed that 90% CIs for the geometric mean ratios of IBI301/rituximab for PK endpoints (AUC_0-inf_, AUC_0-t_ and *C*_max_) all fell within the predefined bioequivalence margin of 0.8–1.25. In addition, the PK curves of the two drug concentrations were almost the same. These data collectively provided potent evidence that IBI301 was PK bioequivalent to the reference drug in NHL. Such similarities in PK have also been observed in other comparative studies on rituximab biosimilars in patients with different NHL subtypes, including CT-P10 and GP2013 in FL^20–22^ and RTXM83 in DLBCL^23^. Moreover, the PK profile of Reditux, a rituximab biosimilar, was comparable to historical reports of rituximab in DLBCL^24^.

Based on the PD results, the two groups showed a markedly decreased value of CD19^+^ and CD20^+^ cells 72 h after infusion, indicating a rapid depletion of peripheral B cells, and this was similar to a phase I dose-escalation trial of IBI301^14^. Likewise, the PD results were consistent with previous data from studies on other rituximab biosimilars in patients with NHL^21–24^.

A meta-analysis demonstrated no significant differences in AEs between rituximab and its biosimilars in RA and NHL^25^. In the present study, the overall incidence of TEAEs in the SS was the same in the IBI301 and rituximab groups. Importantly, no treatment-related deaths were observed in both groups, and the incidences of AEs resulting in discontinuation and withdrawal were both low (< 2.5%).

For TRAEs of grade 3 or above, IBI301 seemed to cause a slightly higher occurrence rate compared with rituximab. However, most of them were not related to the study drug based on the judgment of investigators, and recovered after symptomatic treatment or observation, without any sequelae. Moreover, the most frequent TRAEs of grade 3 or worse were abnormal laboratory tests such as decreased neutrophil count and decreased WBC count. Consistent with the findings of the present study, a decreased neutrophil count was also the most frequent grade 3 or 4 TEAE in a previous study on CT-P10 in low-tumour-burden FL^20^. Since rituximab plays a therapeutic role by inducing the apoptosis of CD20-positive cells and the activation of complement-dependent and antibody-dependent cell cytotoxicity^26^, it is rarely possible that rituximab or its biosimilar would cause decreased neutrophil count and decreased WBC count. In contrast, chemotherapy generally results in myelosuppression reflected by febrile neutropenia (FN), and low absolute neutrophil count and absolute lymphocyte count are two risk factors for FN^27^. As previous treatment regimens (including the treatment duration) were not considered as stratification factors in randomization in the present study, some bias between the two treatment groups might exist regarding chemotherapy, especially differences in the time interval between the last anti-tumour therapy and the first dose of study drug, which might influence the incidence of AEs. In the present study, the aforementioned median time interval in the IBI301 group was 177.0 days (range: 38–1,490), lower than that in the rituximab group (194.0 days, range: 31–1804). Thus, the decreased neutrophil count and decreased WBC count might be associated with the administration of chemotherapy prior to the study drug. In addition, the difference in occurrence rate of haematological toxicity might also be due to variance in previous anti-tumour treatments, such as R ± other chemotherapy (3.4% vs. 6.7%) and unknown treatment (5.7% vs. 2.2%) between IBI301 group and rituximab group (Supplementary Table S2). Notably, the retrospective analysis after unblinding in the present study found that among the participants experiencing the aforementioned AEs of grade 1–2, more participants in the rituximab group received immunostimulants (including G-CSF) compared with those in the IBI301 group (80.0% vs. 44.4%). Moreover, in participants who did not receive immunostimulants (*n* = 80 in the IBI301 group, and *n* = 81 in the rituximab group), the occurrences of the aforementioned AEs were similar between the two treatment groups (IBI301 vs. rituximab): total decreased neutrophil count (21.3% vs. 21.2%), total decreased WBC count (28.8% vs. 27.2%), the grade 3 or 4 decreased neutrophil count (5% vs. 3.7%), and grade 3 or 4 decreased WBC count (2.5% vs. 1.2%), without a statistically significant difference. Collectively, the slightly higher incidence of the aforementioned hematologic toxicities might be related to chemotherapy, instead of IBI301. Overall, the safety profile in the two groups was at a comparable level.

ADAs are of particular interest in biological products because they can affect the efficacy and safety of the drug^28^. In the present study, the incidence of ADA was low in both groups after drug administration (both < 6%), consistent with previously published data on rituximab^29^. As the immunogenicity is product specific, it is not advisable to compare the proportion of patients who developed ADA to other products.

Patient access to biological therapies may be severely limited, especially in low-income countries^30^, but the availability of an effective and safe biosimilar can increase the access to effective therapies, such as rituximab against CD20^+^ B-cell lymphoma.

This study had several limitations. First, as a PK bioequivalence comparative study, parameters for PK, PD, safety, and immunogenicity, but not for clinical efficacy, were evaluated. Second, the sample size was relatively small for safety analysis. In addition, the follow-up was short for safety and immunogenicity assessment. However, the efficacy and safety will be assessed in the following phase 3 study with a larger sample size and longer follow-up.

In summary, IBI301 was PK bioequivalent to rituximab in patients with CD20^+^ B-cell lymphoma who achieved a CR/CRu after standard treatment. Moreover, the PD, safety, and immunogenicity profiles of IBI301 and rituximab were at comparable levels.

## Supplementary information

Supplementary Information.

## Data Availability

All data analysed in the study are provided in the manuscript.
