# Supplementary material for: Pharmacokinetics and safety of IBI301 versus rituximab in patients with CD20+ B-cell lymphoma: a multicenter, randomized, double-blind, parallel-controlled study
Source: Sci Rep. 2020 Jul 15;10:11676. doi: 10.1038/s41598-020-68360-0 (PMC7363910; doi:10.1038/s41598-020-68360-0)
Supplement: Supplementary file 1 — Supplementary Information. [file 41598_2020_68360_MOESM1_ESM.pdf]

Pharmacokinetics and safety of IBI301 versus rituximab in patients with CD20$^+$ B-cell lymphoma: A multicenter, randomized, double-blind, parallel-controlled study

Running title: Bioequivalence study of IBI301 to rituximab

Bo Jiang [1†], Xiaoyan Ke [2†], Qingyuan Zhang [3†], Wei Xu [4], Hang Su [5], Jie Huang [6], Mingzhi Zhang [7], Huaqing Wang [8], Chuan Jin [9], Jun Zhu [10], Li Liu [11], Zhen Cai [12], Xielan Zhao [13], Jianfeng Zhou [14], Xiaohong Zhang [15], Jing Liu [16], Hui Zhou [17], Jie Yu [17], Xing Sun [17], Junyuan Qi [1], Lugui Qiu [18*]

1 Phase I Clinical Trial Unit, Institute of Hematology & Blood Diseases Hospital, Chinese Academy of Medical Sciences;
2 Hematology Department, Peking University Third Hospital;
3 Oncology Department, The Affiliated Cancer Hospital of Harbin Medical University;
4 Hematology Department, Jiangsu Province Hospital;
5 Department of lymphoma/head and neck oncology, The Fifth Medical Center of PLA General Hospital;
6 Hematology Department, West China Hospital Sichuan University;
7 Oncology Department, The First Affiliated Hospital of Zhengzhou Medical University;
8 Oncology Department, Tianjin Union Medical Center Nankai University Affiliated Hospital;
9 Forth Medical Department, The Affiliated Cancer Hospital of Guangzhou Medical University;
10 Key Laboratory of Carcinogenesis and Translational Research (Ministry of Education), Department of Lymphoma, Peking University Cancer Hospital & Institute;
11 Hematology Department, Tangdu Hospital, the Medical University of Air Forces;
12 Bone marrow transplantation center, The First Affiliated Hospital of Zhejiang University School of Medicine;
13 Hematology Department, Xiangya Hospital Central South University;
14 Hematology Department, Tongji Medical College Huazhong University of Science & Technology;
15 Hematology Department, The Second Affiliated Hospital of Zhejiang University School of Medicine;
16 Hematology Department, The Third Xiangya Hospital of Central South University;
17 Innovent Biologics, Inc.;
18 Lymphoma Center, Institute of Hematology & Blood Diseases Hospital, Chinese Academy of Medical Sciences

[†]These authors contributed equally to this work.

[*]Corresponding author: Lugui Qiu, Lymphoma Center, Institute of Hematology & Blood Diseases Hospital, Chinese Academy of Medical Sciences
Tel: 13821266636; Email: qiulugui@outlook.com

SUPPLEMENTARY MATERIAL

Inclusion criteria

The major inclusion criteria were: (1) histologically or cytologically confirmed $CD20^+$ B-cell lymphoma patients and could benefit from the study judged by the investigator; (2) the patients achieved a CR/CRu after standard treatment according to the standard response criteria for NHL (International Working Group 1999 criteria), and the diagnosis of CRu was confirmed by enhanced computed tomography or magnetic resonance imaging examination; (3) aged between 18 and 65 years; (4) Eastern Cooperative Oncology Group performance status (PS) 0-1; (5) had at least 6 months of life expectancy as evaluated by investigators; (6) had adequate organ function, including absolute neutrophil count ≥1.5 $\times 10^9$/L, hemoglobin ≥80 g/L, platelet count ≥75 $\times 10^9$/L, total bilirubin ≤1.5 × upper limit of normal (ULN), aspartate aminotransferase and alanine aminotransferase ≤2.5 × ULN, and serum creatinine ≤1.5 × ULN; (7) previous anti-tumour therapy had been terminated (for at least 4 weeks for chemotherapy, at least 16 weeks for rituximab or other targeted therapy, and at least 4 weeks for radiotherapy); and adverse events related to chemotherapy, surgery, radiation or target therapy with clinical significance had been alleviated to grade ≤1; (8) effective contraceptive methods were conducted for both male and female patients during the whole study period and within 12 months after drug discontinuation.

Exclusion criteria

The exclusion criteria were patients: (1) dropped out of any interventional clinical study within 28 days prior to enrollment; (2) received blood transfusion, erythropoietin, granulocyte colony stimulating factor, or granulocyte macrophage colony stimulating factor within 14 days before enrollment; (3) inoculated or planned to inoculate with live-virus vaccine within 28 days before enrollment; (4) underwent or planned to undergo major surgery within 28 days before enrollment, or with unhealed surgical wound; (5) received high-dose corticosteroids (hydroprednisone >10 mg/day or equivalent dose) within 28 days prior to enrollment; (6) received or planned to undergo allogeneic hematopoietic stem cell transplantation; received or planned to undergo autologous hematopoietic stem cell transplantation within 3 months before baseline (patients who have undergone autologous hematopoietic stem cell transplantation 3 months before baseline can be enrolled upon confirmation with the sponsor's medical examiner); (7) had history of gastrointestinal perforation and/or fistula disease within 6 months before enrollment; (8) with allergy to monoclonal antibody (mAb) therapy or rituximab; (9) used rituximab or other anti-CD20 mAb drugs within 4 months before screening; (10) had residual peripheral rituximab concentration of >24 μg/ml at screening (they were allowed a re-selection after a washout period when the concentration of peripheral rituximab was lower than 24 μg/mL); (11) with chronic lymphocytic leukemia/small lymphocytic lymphoma; (12) were positive for human immunodeficiency virus antibody; (13) were positive for hepatitis C virus antigen or antibody; (14) had acute or chronic hepatitis B virus infection, including those with positive hepatitis B virus (HBV) surface antigen; if patients were with negative HBV surface antigen but positive HBV core antibody, the enrollment was allowed only when

HBV DNA level (viral load) was below the minimum lower limit (1.0×10^3 copies /mL); or (15) with serious diseases or conditions, including but not limited to: (i) diseases known to be associated with human immunodeficiency virus infection or acquired immunodeficiency syndrome; (ii) severe infections that were active or clinically poorly controlled; (iii) asthma or interstitial lung disease or severe chronic obstructive pulmonary disease; (iv) had myocardial infarction, unstable angina pectoris or had received cardiovascular intervention/surgery or had congestive heart failure within 6 months prior to enrollment (CHF; NYHA II-IV), or had symptomatic or poorly controlled arrhythmias; (v) arterial systolic pressure ≥140 mmHg or diastolic blood pressure ≥90 mmHg after treatment; (vi) acute or chronic hypotension (systolic blood pressure <90 mmHg or diastolic blood pressure <60 mmHg); (vii) diabetes responding to medicine poorly; (viii) body mass index ≥28 kg/m^2; (ix) thyroid dysfunction (Thyroid Stimulating Hormone lower than the lower limit of normal values or higher than the ULN, with clinical significance as judged by the investigators); (x) medical history of central nervous system invasion or cranial neuropathy; (xi) history of toxic epidermal necrolysis or Stevens-Johnson syndrome; (xii) RA, granulomatous vasculitis or microscopic polyangiitis; (xiii) intestinal obstruction, or history of inflammatory bowel disease or extensive bowel resection (partial colectomy or extensive small bowel resection with chronic diarrhea), Crohn's disease, ulcerative colitis, or chronic diarrhea; (xiv) history of other malignant diseases, with the exception of: basal cell carcinoma or cutaneous squamous cell carcinoma and/or cervical carcinoma in situ, or other hematological and solid tumours that have been cured and have no evidence of recurrence for at least 3 years prior to enrollment; or (xv) history of other conditions, acute or chronic diseases, and mental illness or laboratory abnormalities that may lead to increased risks associated with study participation or study drug administration, or interference with the interpretation of study results, or the patients were listed as ineligible to participate in this study by the investigators.

Immunogenicity

Samples were collected from all subjects during the screening period, and at 14±1 d, 28±2 d, 42±3 d, 70±3 d, and 91±3 d after infusion. For each sampling, 5 mL of whole blood were collected with a coagulation vacuum blood collection tube, and serum was separated. The anti-drug antibody (ADA) analysis used electrochemiluminescence method consisting of three components: screening assay, confirmation assay and titration assay. All samples were subject to preliminary screening assay, and those with a high value above or equal to the cut point were considered as suspected ADA-positive samples. The suspected ADA-positive samples underwent further confirmatory assay, which included dosing and non-dosing-parallel test.

Compared to the non-dosing tested samples, the sample with a true positive antibody response would produce a significant inhibitive response (expressed as a percentage) after drug administration (IBI301 or rituximab). The positive samples subsequently underwent titer assay, and were diluted to the extent at least one sample had the value below the plate-specific titer threshold, which was prepared for the semi-quantitative analysis. The confirmed ADA-positive serum samples were tested for the neutralizing antibodies (NAb) analysis.

The NAb assay was based on the principle of complement-dependent cytotoxicity assay. The CellTiter-Glo assay (Promega, Madison, WI, USA) was used to detect NAb situation of IBI301 or the primary drug in human serum.

Reference

1. Cheson, B. D. *et al*. Report of an international workshop to standardize response criteria for non-Hodgkin's lymphomas. NCI Sponsored International Working Group. J Clin Oncol. 17, 1244 (1999).

Supplementary Table S1. Treatment-emergent adverse events in the safety set

	IBI301 (*n* = 89), *n* (%)		Rituximab (*n* = 91), *n* (%)	
	Any grade	Grade ≥3	Any grade	Grade ≥3
**TEAE**	75 (84.3)	24 (27.0)	76 (83.5)	12 (13.2)
Decreased WBC count	30 (33.7)	6 (6.7)	29 (31.9)	3 (3.3)
Decreased neutrophil count	25 (28.1)	9 (10.1)	25 (27.5)	5 (5.5)
Upper respiratory infection	21 (23.6)	1 (1.1)	12 (13.2)	0
Decreased lymphocyte count	13 (14.6)	2 (2.2)	8 (8.8)	2 (2.2)
Elevated ALT level	12 (13.5)	1 (1.1)	9 (9.9)	1 (1.1)
Hypertriglyceridemia	11 (12.4)	3 (3.4)	4 (4.4)	0
Elevated blood triglyceride level	9 (10.1)	0	6 (6.6)	0
Hypercholesterolemia	6 (6.7)	0	1 (1.1)	0
Elevated AST level	6 (6.7)	0	5 (5.5)	0
Elevated hemobilirubin level	5 (5.6)	0	8 (8.8)	0

Date are n (%). Treatment-emergent adverse events (TEAEs) occurring in ≥5% and grade ≥3 AEs occurring in ≥1% of patients in either group are listed. ALT, Alanine aminotransferase; AST, aspartate aminotransferase; WBC, white blood cell.

Supplementary Table S2. Previous treatment prior to the study in two groups

	IBI301 (*n* = 89), *n* (%)	Rituximab (*n* = 91), *n* (%)	All (*n* = 180), *n* (%)	*P*
Received prior anti-tumour drug therapy	87 (97.8)	89 (97.8)	176 (97.8)	>0.99
Previous treatment regimens*				
ASCT	2 (2.3)	1 (1.1)	3 (1.7)	
R+DA-ECHOP	1 (1.1)	3 (3.4)	4 (2.3)	
R+DHAP	5 (5.7)	6 (6.7)	11 (6.3)	
R+ICE	7 (8.0)	3 (3.4)	10 (5.7)	
R-ESHAP	4 (4.6)	3 (3.4)	7 (4.0)	
R-FC/FMD	5 (5.7)	3 (3.4)	8 (4.5)	
R-GDP/GMOX	4 (4.6)	4 (4.5)	8 (4.5)	
R±CHOP±E	81 (93.1)	84 (94.4)	165 (93.8)	
R±Hyper CVAD	4 (4.6)	1 (1.1)	5 (2.8)	
R±Other chemotherapy	3 (3.4)	6 (6.7)	9 (5.1)	

	IBI301<br>(*n* = 89), *n* (%)	Rituximab<br>(*n* = 91), *n* (%)	All<br>(*n* = 180), *n* (%)	*P*
Unknown	5 (5.7)	2 (2.2)	7 (4.0)	
Other adjuvant therapy	3 (3.4)	3 (3.4)	6 (3.4)	
Other chemotherapy	20 (23.0)	12 (13.5)	32 (18.2)	
Lumbar puncture intrathecal injection	2 (2.3)	0	2 (1.1)	
Pretreatment	2 (2.3)	2 (2.2)	4 (2.3)	
Radiotherapy history	14 (15.7)	11 (12.1)	25 (13.9)	0.52

ASCT: autologous stem cell transplantation; R: rituximab; CHOP: cyclophosphamide, doxorubicin, vincristine and prednisone; E: etoposide; DA-ECHOP: dose-adjusted ECHOP; DHAP: dexamethasone, cytarabine, and cisplatin; ICE: ifosfamide, carboplatin, etoposide; ESHAP: etoposide, methyl prednisolone, cisplatine, cytarabine. FC: fludarabine and cyclophosphamide; FMD: fludarabine, mitoxantrone and dexamethasone; GDP: gemcitabine, dexamethasone and cisplatin; GMOX: gemcitabine, and oxaliplatin; hyper CVAD: hyper-fractionated cyclophosphamide, adriamycin, vincristine, and dexamethasome. *: As a patient could receive more than one line of therapies, and thus the sum of patients receiving the previous treatment regimens were more than 87.

Figure legends

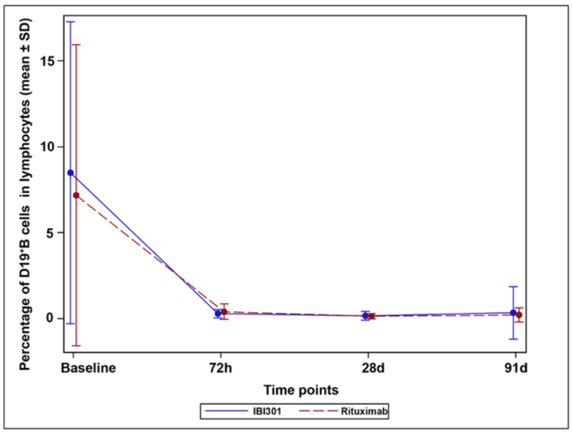

Supplementary Figure S1. Absolute value of peripheral CD19^{+} B cells of the patients in pharmacokinetic set. Error bars are standard deviations.

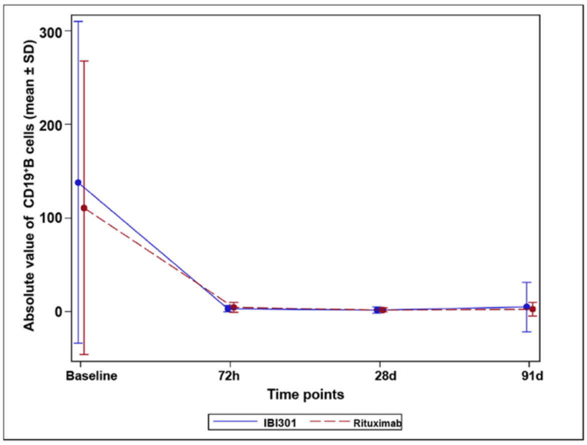

Supplementary Figure S2. Percentage of peripheral CD19^{+} B cells of the patients in pharmacokinetic set. Error bars are standard deviations.

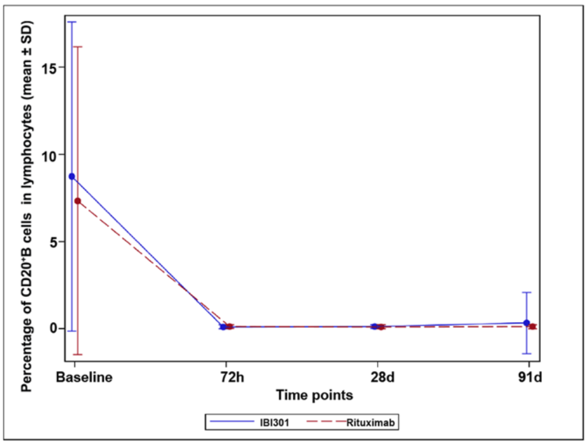

Supplementary Figure S3. Absolute value of peripheral CD20^{+} B cells of the patients in pharmacokinetic set. Error bars are standard deviations.

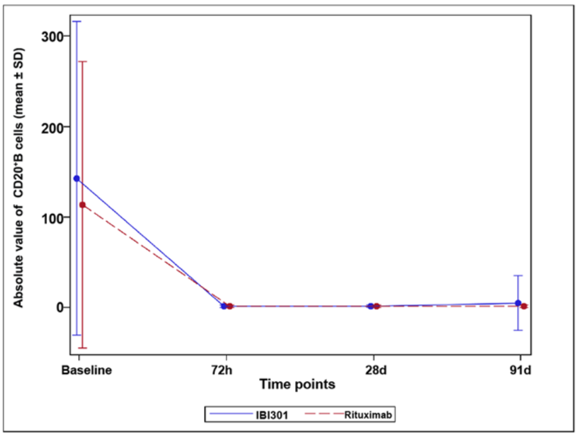

Supplementary Figure S4. Percentage of peripheral CD20$^+$ B cells of the patients in pharmacokinetic set. Error bars are standard deviations.
